# Opioid use in the era of COVID-19: a multifaceted study of the opioid epidemic in Canada

**DOI:** 10.3389/fphar.2023.1122441

**Published:** 2023-05-25

**Authors:** Molly Hutchinson, Éric Lavigne, Zachary Patterson

**Affiliations:** ^1^ Faculty of Public Affairs, Carleton University, Ottawa, ON, Canada; ^2^ Air Health Science Division, Health Canada, Ottawa, ON, Canada; ^3^ School of Epidemiology and Public Health, University of Ottawa, Ottawa, ON, Canada; ^4^ Department of Neuroscience, Carleton University, Ottawa, ON, Canada

**Keywords:** harm reduction, opioid, overdose, opioid crisis, COVID-19, public health

## Abstract

**Introduction:** The COVID-19 pandemic has had wide economic, social, and health impacts, and has disproportionately affected individuals who were already vulnerable. Individuals who use opioids have dealt with evolving public health measures and disruptions while also dealing with the ongoing opioid epidemic. Opioid-related mortalities in Canada increased throughout the COVID-19 pandemic, but it is unclear to what extent public health measures and the progression of the pandemic contributed to opioid-related harms.

**Methods:** To address this gap, we used emergency room (ER) visits recorded in the National Ambulatory Care Reporting System (NACRS) between 1 April 2017, and 31 December 2021, to investigate trends of opioid-related harms throughout the pandemic. This study also included semi-structured interviews with service providers in the field of opioid use treatment, to help contextualize the trends seen in ER visits and offer perspectives on how opioid use and services have changed throughout the COVID-19 pandemic.

**Results:** Overall, the number of hospitalizations related to an opioid use disorder (OUD) decreased with progressing waves of the pandemic and with increasing severity of public health measures in Ontario. The rate of hospitalizations related to opioid poisonings (e.g., central nervous system and respiratory system depression caused by opioids) significantly increased with the progressing waves of the pandemic, as well as with increasing severity of public health measures in Ontario.

**Discussion:** The increase in opioid-related poisonings is reflected in the existing literature whereas the decrease in OUDs is not. Moreover, the increase in opioid-related poisonings aligns with the observations of service providers, whereas the decrease in OUD contradicts the trends that service providers described. This discrepancy could be explained by factors identified by service providers, including the pressures on ERs during the pandemic, hesitancy to seek treatment, and drug toxicity.

## 1 Introduction

The opioid epidemic has harmed communities, families, and frequently some of the most vulnerable populations in Canada for decades. Between January 2016 and September 2021, there were 26,690 fatal opioid poisonings recorded, 96% of which were deemed accidental (Canadian Center on Substance Use and Addiction, 2021; Public Health Agency of Canada, 2022). The past few years have been marked with an increase in organized initiatives to combat the opioid crisis. These include: increasing accessibility of naloxone kits and safe consumption sites, offering opioid agonist treatment (OAT), increasing mental health supports, and addressing the stigma around substance use disorders (SUD) by spreading community awareness (Health Canada, 2020; Cheetham et al., 2022).

When the COVID-19 pandemic struck Canada in March 2020, many of the efforts to support individuals using opioids and prevent the rise of accidental opioid-related overdoses were disrupted, closed, or stalled indefinitely (Health Canada, 2020; Joudrey et al., 2021). This abrupt shift, paired with increased stress and isolation caused by the pandemic, contributed to increased opioid use and decreased access to opioid use supports and treatment in Canada (Health Canada, 2020). Moreover, supply chains implicated in the movement of illicit drugs were heavily impacted by border closures and travel restrictions, significantly altering the make-up and predictability of the illicit opioid marketplace (Health Canada, 2020). The COVID-19 pandemic commanded the near-complete attention of many Canadians and pushed other important public health and societal issues out of the spotlight, and therefore out of the immediate public consciousness. As the COVID-19 pandemic continued to take a toll on communities all over the world, the opioid epidemic has been worsening under the radar.

Individuals who use opioids are more likely to develop COVID-19, suffer from comorbid diseases, go untested for COVID-19, live in conditions that make it difficult to socially distance and self-isolate, and suffer from discrimination in the medical system (Bahji et al., 2021). In addition to these vulnerabilities to the COVID-19 virus, the pandemic has had massive impacts on the opioid epidemic itself (Bahji et al., 2021). From April 2020 to March 2021, 22,830 COVID-19 deaths were recorded in Canada (Jackson, 2021). During the same period, 7,224 opioid toxicity deaths were recorded, approximately one-third of the number of COVID-19 deaths (Public Health Agency of Canada, 2022). These opioid-related deaths overwhelmingly occurred in individuals under the age of 60, with 47% occurring in individuals between the ages of 20 and 40 years old (Public Health Agency of Canada, 2022).

**FIGURE 1:**
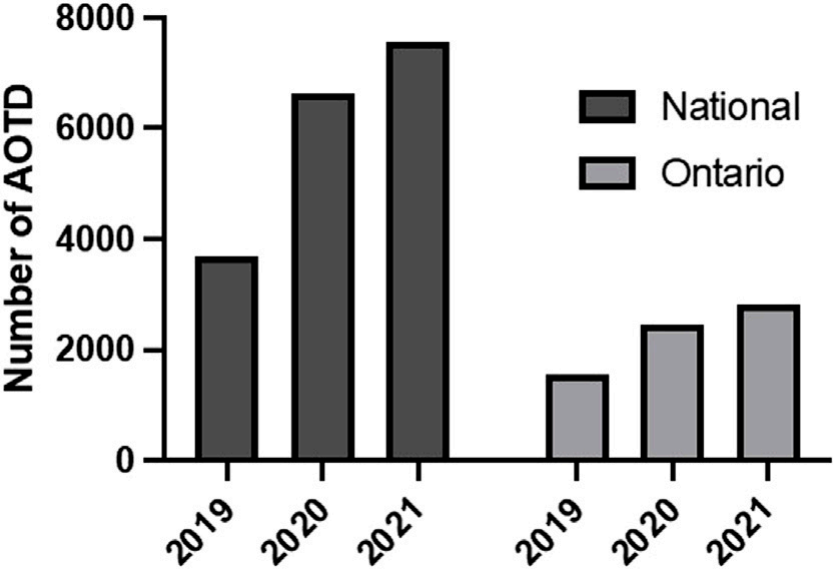
Number of apparent opioid toxicity deaths (AOTD) in Canada and Ontario in 2019, 2020, and 2021 (adapted from the public health agency of Canada, 2022).

Early data emerging on the impact of the COVID-19 pandemic on opioid-related harms clearly shows that the COVID-19 pandemic is correlated with a significant increase in opioid-related morbidity and mortality (Public Health Agency of Canada, 2022; see Figure 1). Over the first 6 months of the pandemic, 1,237 people died in Ontario from opioid-related causes, totaling an additional 17,843 years of life lost compared to the previous 6 months (Gomes et al., 2021). During those first 6 months, the largest increase in opioid-related deaths was seen in individuals aged 23 to 54 (135%), and more specifically in men younger than 35 years old (320%; Gomes et al., 2021). Across all age groups and demographics, emergency medical services (EMS) visits related to opioid use increased by 57% during the first year of the pandemic, and opioid overdoses across all age groups increased by 60% (Friesen et al., 2021). Rural and northern communities, people experiencing homelessness, people living in poverty, incarcerated individuals, and BIPOC (Black, Indigenous and People of Colour) communities experienced disproportionately high increases in overdoses (Friesen et al., 2021). Youth and young adults (29 years old and younger) have also been particularly vulnerable to increased opioid overdoses during the pandemic due to increased access to prescription drugs intended for family members, the tendency of young people to cope with negative emotions with high-risk behavior, and the inherent vulnerability of the youth stages of biopsychosocial development (Jayasinha et al., 2020).

In addition to mortality rates and number EMS calls, emergency room (ER) visits related to opioid use can reveal trends in opioid-related harms. There has been a steady increase in ER visits for opioid-related reasons since the beginning of 2020, with a rate of 57.7 ER visits per 100,000 people at the beginning of 2020 and 120.3 per 100,000 by mid-2021 (Public Health Ontario, 2022b). A study from Los Angeles used emergency room visits to track the impact of the COVID-19 pandemic and related lockdowns on opioid-related harms and found that uninsured and racialized individuals were the most heavily impacted (Johnson et al., 2021).

The COVID-19 pandemic, with its significant disruptions and challenges, has worsened the opioid epidemic. There exist many theories as to how changes to the illicit drug marketplace, individual stress, and decreased access to opioid use supports and services have impacted opioid-related harms during the pandemic; however, there has been minimal analysis of how public health measures that were put in place to combat the spread of COVID-19 have impacted the opioid epidemic. For example, the pandemic has resulted in capacity restrictions, the movement of services online or over the phone, limited social gatherings, and altered social services, which can now be studied more directly for their impact on opioid-related harms. Moreover, there has not been significant analysis of how different opioid-related harms changed in response to the pandemic. The objective of this study is to identify how policy decisions and the cumulative effect of the pandemic impacted rates of recorded opioid-related poisonings and opioid use disorders (OUD), with the goal of providing evidence to inform the consideration of people who use drugs (PWUD) in policy decisions related to future pandemic and non-pandemic policy.

## 2 Materials and methods

### 2.1 Study design

This study used a retrospective analysis of emergency room (ER) visits for opioid-related reasons between 1 April 2017, and 31 December 2021. To help contextualize the quantitative data analysis, this study also included semi-structured interviews (conducted between May 2022 and August 2022) with service providers in the fields of opioid use and opioid poisoning treatment who provided services during the COVID-19 pandemic in Toronto, Ontario, Canada.

### 2.2 Data sources

#### 2.2.1 National ambulatory care reporting system (NACRS)

Anonymized NACRS data was obtained from Health Canada, which includes records of ER visits from participating hospitals across Canada. The provinces and territories included in this data set are Alberta, New Brunswick, Nova Scotia, Ontario, P.E.I, Saskatchewan, and Yukon. ER visits associated with an OUD or opioid-related poisoning as at least one of the reasons for the visit were captured in the data set. Opioid-related reasons for presenting to the ER were determined using the International Statistical Classification of Diseases and Related Health Problems (ICD) codes that pertain to opioid-related poisonings and OUDs. The following ICD codes were used to identify hospital visits related to opioid poisonings and OUDs: F11.X (F11.0, F11.1, F11.2, F11.3, F11.4, F11.5, F11.6, F11.7, F11.8, F11.9), and T40.X (T40.2, T40.20, T40.21, T40.22, T40.23, T40.28, T40.3, T40.4, T40.40, T40.48, T40.60) (ICD-10-CM/PCS Transition, 2019; ICD-10 Codes Lookup *, ICD-10-CM Codes Search - Codify by AAPC*, n.d.; Public Health Ontario, 2023; see Table 1).

**TABLE 1:** ICD code descriptions (*ICD—ICD-10-CM—international classification of diseases,* ICD-10-CM/PCS Transition, 2019; ICD-10 Codes Lookup *, ICD-10-CM Codes Search—Codify by AAPC*, n.d.; Public Health Ontario, 2023).

ICD code	Description	ICD code	Description
F11.0	Opioid related disorders	T40.2	Poisoning by codeine and derivatives
F11.1	Harmful opioid use	T40.21	Poisoning by morphone
F11.2	Opioid dependence	T40.22	Poisoning by hydromorphone
F11.3	Opioid withdrawal	T40.23	Poisoning by oxycodone
F11.4	Opioid withdrawal with delirium	T40.28	Poisoning by other opioids
F11.5	Opioid-related psychotic behaviour	T40.3	Poisoning by methadone
F11.6	Opioid-related amnesic syndrome	T40.4	Poisoning by fentanyl and derivatives
F11.7	Opioid-related residual and late-onset psychotic disorder	T40.48	Poisoning by other synthetic narcotics
F11.8	Opioid-related mental and behavioural disorder	T40.6	Poisoning by other and unspecified narcotics
F11.9	Opioid use, unspecified		

#### 2.2.2 Semi-structured interviews

This study also employed semi-structured interviews with four participants who are service providers in the field of opioid use treatment in Ontario. The positions of the individuals interviewed include peer support program executive director, peer support worker, harm reduction program coordinator, and women’s shelter employee, and their respective contributions are labeled throughout the results section. Each semi-structured interview with service providers was approximately 45 min long and included questions pertaining to the illicit opioid supply, access to OUD and opioid overdose treatment, the prevalence of OUDs, and opioid-related mortality. Interviews were transcribed by hand and then underwent validity checks. Audio files were deleted once the transcripts were validated, and only the de-identified transcripts were kept.

### 2.3 Participants

#### 2.3.1 Patients

Participants in the quantitative analysis were individuals presenting to an ER in Canada with at least one opioid-related concern (as identified by ICD codes). The NACRS data on ER visits between 1 April 2017, and 31 December 2021 in Canada included the province/territory of the medical facility visited, the biological sex of the patient (M/F), the year of birth of the patient, the age of the patient, whether the patient presented with an opioid-related poisoning, whether the patient presented with an OUD, and the top six reasons for the visit, listed as the “main problem,” “other problem 1,” “other problem 2,” “other problem 3,” “other problem 4,” and “other problem 5.”

#### 2.3.2 Service providers

Participants in the qualitative analysis comprised of service providers in Ontario working in the field of OUD treatment or opioid use harm reduction before and during the COVID-19 pandemic. Interview participants were recruited using an email invitation and snowball recruitment. Eligibility to participate in semi-structured interviews included being a service provider in the field of opioid use treatment and working with individuals who used opioids or were supporting someone who used opioids. Participants were also required to speak English and be at least 18 years old.

### 2.4 Data analysis

#### 2.4.1 Quantitative analysis

To assess the impacts of the COVID-19 pandemic on opioid-related harms, the NACRS data was organized and coded to allow for an analysis of opioid-related harms throughout waves of the pandemic, stages of provincial public health measures (only in Ontario), and the Canada Emergency Response Benefit (CERB).

Each ER visit was coded for occurring before the pandemic (i.e., occurring before March 2020) or the specific wave of the pandemic in which it occurred. Waves of the pandemic have been characterized in the literature as periods of time with peaks in COVID-19 cases and/or the presence of a particular COVID-19 variant and are therefore relevant for understanding how different periods of the pandemic and the progression of the pandemic over time have impacted opioid-related harms. Since there was no single authority on the start and end dates for each wave of the pandemic to our knowledge, the timeline for each wave of the pandemic was determined using a combination of news articles, publications from public health agencies, and literature on the COVID-19 pandemic. Although it was possible to find dates for waves 1, 2, 3, and 4 published by Public Health Ontario, which was determined to be the most authoritative source available, the fifth wave was recent at the time of the research and was not as clearly defined. Therefore, a combination of news articles and public health statements were used to estimate the start date of the fifth wave. The waves that were identified were broken down as follows: wave 1 from 26 February 2020 to 31 August 2020, wave 2 from 1 September 2020 to 28 February 2021, wave 3 from 1 March 2021 to 31 July 2021, wave 4 from 1 August 2021 to 16 December 2021, and wave 5 starting 17 December 2021 (Public Health Ontario, 2022a; Smart, 2021; see Tables 2, 3).

**TABLE 2:** Number of daily and total visits for an opioid-related poisoning and/or OUD in NACRS data by wave nationally. Waves were defined using a combination of news articles, publications from public health agencies, and literature on the COVID-19 pandemic. The average number of daily visits for an opioid-related reason was not significantly associated with waves nationally (*p* = 0.096).

Wave	Start date	End date	Number of days	Number of visits	Visits per day
0	Apr. 1 2017	Feb. 25 2020	1,061	106,379	100.26
1	Feb. 26 2020	Aug. 31 2020	188	25,647	136.42
2	Sep. 1 2020	Feb. 28 2021	181	24,912	137.64
3	Mar. 1 2021	Jul. 31 2021	153	22,230	145.29
4	Aug. 1 2021	Dec. 16 2021	138	21,247	153.96
5	Dec. 17 2021	Dec. 31 2021	15	2082	138.80
Total			1736	202,497	

**TABLE 3:** Number of daily and total visits for an opioid-related poisoning and/or OUD in Ontario NACRS data by wave. Waves were defined using a combination of news articles, publications from public health agencies, and literature on the COVID-19 pandemic. The average number of daily visits to the ER for an opioid-related reason was strongly positively associated with the waves of the pandemic in Ontario (adj. R^2^ = 0.730, *p* = 0.019, β = 0.885).

Wave	Start date	End date	Number of days	Number of visits	Visits per day
0	Apr. 1 2017	Feb. 25 2020	1,061	52,797	49.76
1	Feb. 26 2020	Aug. 31 2020	188	11,204	59.60
2	Sep. 1 2020	Feb. 28 2021	181	13,263	73.28
3	Mar. 1 2021	Jul. 31 2021	153	13,277	86.78
4	Aug. 1 2021	Dec. 16 2021	138	12,260	88.84
5	Dec. 17 2021	Dec. 31 2021	15	1,223	81.53
Total			1736	104,024	

In addition to waves of the pandemic, Ontario ER visits were also coded for the stage of provincial public health measures when they occurred. Stages of public health measures were based on the implementation of distinct sets of measures such as stay-at-home orders, lockdown measures, stages of reopening as defined in the Ontario government’s *A Framework for Reopening Our Province*, and steps for reopening as defined in the Ontario government’s *Roadmap to Reopen* (Office of the Premier, 2020; Office of the Premier, 2021). Stages of the pandemic that occurred multiple times, such as the multiple different stay-at-home orders that were instated over the course of the pandemic, were combined to allow for an analysis of the relationship between the type of public health measure and opioid-related harms. The stages were only coded for Ontario ER visits due to the provincial nature of the public health measures that were implemented. Public health stages were organized ordinally for a regression analysis and were ordered from the least strict public health measures to the strictest public health measures, based on an estimate of strictness from available information. The timeline and descriptions of these provincial public health measures were compiled using news articles, publications from public health agencies, and literature on the COVID-19 pandemic (see Tables 4, 5).

**TABLE 4:** Principles and policies of Ontario’s public health stages (Hawley, 2020; Dainton & Hay, 2021; Office of the Premier, 2021; Tsekouras, 2021; Zuber, 2021).

Stage	Strictness of regulations
Stay-at-home order	- Only permitted to leave home for essential purposes
Lockdown	- Opening select workplaces, allowing essential gatherings with limited number of people, opening some outdoor spaces, continued protections for vulnerable populations
Stage 1	- Opening more workplaces, opening more public spaces, allowing some larger public gatherings, continued protections for vulnerable populations
Stage 2	- Opening more workplaces, opening more public spaces, allowing some larger public gatherings, continued protections for vulnerable populations
Stage 3	- Opening all workplaces, relaxing restrictions on public gatherings, continued protections for vulnerable populations
Step 1	- When at least 60% of Ontario adults have received at least one dose of the vaccine and if public health indicators indicate that the province can move safely into the next step
	- Resuming small outdoor gatherings and permitting retail with restrictions
Step 2	- When at least 70% of Ontario adults have received at least one dose and 20% have two doses and there are positive trends in public health and health system indicators
	- Expanding outdoor activities and resuming small indoor services where face coverings are worn
Step 3	- When 70%–80% of Ontario adults have received at least one dose and 25% of adults have two doses and positive trends in public health and health system indicators continue
	- Increased access to indoor settings with some restrictions on large gatherings where masks cannot be worn, including indoor sports and recreational fitness; indoor dining, museums, art galleries and libraries, and casinos and bingo halls

**TABLE 5:** Number of Daily and Total Visits for an Opioid-Related Poisoning and/or OUD in Ontario NACRS Data by Stage. Stages of public health measures were based on the implementation of distinct sets of measures such as stay-at-home orders, lockdown measures, stages of reopening as defined in the Ontario government’s *A Framework for Reopening Our Province*, and steps for reopening as defined in the Ontario government’s *Roadmap to Reopen* (Office of the Premier, 2020; Office of the Premier, 2021). Stages of the pandemic that occurred multiple times, such as the multiple different stay-at-home orders that were instated over the course of the pandemic, were combined to allow for an analysis of the relationship between the type of public health measure and opioid-related harms. The average number of daily visits for an opioid-related reason was not significantly associated with stages of the pandemic in Ontario (*p* = 0.830).

Stage	Number of days	Number of visits	Visits per day
Prepandemic	1,082	54,334	50.22
Step 3	156	13,856	88.82
Step 2	16	1,507	94.19
Step 1	19	1,688	88.84
Stage 3	85	5,630	66.24
Stage 2	79	5,138	65.04
Stage 1	24	1,403	58.46
Lockdown	164	11,442	69.77
Stay-at-home order	111	9,026	81.32
Total	1736	104,024	

Stay-at-home orders were characterized by government policy that required individuals to only leave their homes for essential purposes (Tsekouras, 2021). Lockdowns are a more general label for periods of time when only select workplaces were open, essential gatherings were permitted with limitations on the number of people, some outdoor spaces were open, and there were ongoing significant protections for vulnerable individuals (Dainton & Hay, 2021; Zuber, 2021; Dmetrichuk et al., 2022). The stages of the pandemic were defined within *A Framework for Reopening Our Province* from the government Ontario. Stage 1 involved opening more workplaces and public spaces, and allowing some larger public gathering (Hawley, 2020). Stage 2 involved even more workplaces and public spaces opening and even more large gatherings (Hawley, 2020). Stage 3 was defined as opening all workplaces and relaxing restrictions on public gatherings, while still protecting vulnerable individuals (Hawley, 2020). The steps of the *Roadmap to Reopen* came after, and were three steps of continued reopening that advanced based on the number of vaccinated individuals and trends in COVID-19 cases, and were focused on relaxing restrictions on services and gathering sizes (Office of the Premier, 2021; Ontario Office of the Premier, 2021).

Finally, to assess how the rates of opioid-related harms changed depending on the implementation of the Canadian Emergency Response Benefit (CERB), a timeline of CERB was taken from the Canadian Revenue Agency. CERB provided individuals residing in Canada with temporary financial aid during the early part of the COVID-19 pandemic and payments were provided from 6 April 2020, to 6 December 2020 (D’Amore & Goldfinger, 2020; Service Canada, 2020). Eligibility depended on applicants having resided in Canada since they were 15 years old, having earned a minimum of $5,000 before tax in the preceding 12 months, not having voluntarily quit their job, and either having had work hours reduced because of COVID-19, stopped working because of COVID-19, been unable to work during COVID-19 due to caring for someone else, or been paid regular employment insurance for at least a week since 29 December 2019 and used up the benefits (Canada Revenue Agency, 2020).

Statistical analyses were performed using SPSS (SPSS, Chicago, Illinois, United States). Prior to analysis, several validity checks were performed to ensure quality of data. Data cleaning was performed to remove all individuals with a date of birth or age that did not make sense. For example, patients with ages listed above 100 years, patients listed as 0 years old, patients with an unknown birth age unit, and patients with their birth years listed as 9,999 were all removed since all these patients did not have age data that was internally consistent. Daily averages for each of the dependent variables were calculated (e.g., presence of an OUD, presence of an opioid-related poisoning, etc.) to be able to look at trends over time and compare daily averages of opioid-related harms between waves, stages, and phases of CERB. Linear regressions were used to examine the relationship between the independent and dependent variables. Significance was determined at *p* < 0.05. The sum of square of the regressions (SSR) were checked for statistical significance, as were the F-values. Moreover, assumptions of normality, homoscedasticity, and linearity were tested in SPSS and were met for all regressions included in the analysis. Cook’s distance was used to check for bias from influential cases and was less than 1 for all regressions.

#### 2.4.2 Qualitative analysis

The semi-structured interviews were analyzed using a thematic analysis as described by Braun & Clark (2006). Briefly, the thematic analysis included four distinct steps: transcription, coding, analysis, and the written report, with an overall attention to the internal consistency of the analysis done on the data set (Braun & Clark, 2006). Interviews were transcribed by hand and were checked against recordings for accuracy (Braun & Clark, 2006). Next, themes were determined by systematically combing through the data and building relevant and internally coherent themes, followed by an analysis using the themes and relevant excerpts (Braun & Clark, 2006). Finally, the written report includes a description of the active process undergone for quantitative analysis (Braun & Clark, 2006).

### 2.5 Ethics clearance

The use of the NACRS data was authorized by the Carleton University Research Ethics Board B and through a data sharing agreement between Health Canada and the Canadian Institute for Health Information (CIHI). The semi-structured interviews were authorized by the Carleton University Research Ethics Board A.

## 3 Results

This data set only includes individuals who presented to a participating ER with an opioid-related concern (as assessed by at least one opioid-related ICD code being associated with that visit). This means that all patients captured in the results section below had at least one opioid-related issue, but each patient could have been admitted for one or more opioid-related poisoning and OUD related issue. Each ER visit gets assigned an ICD code for the primary reason for the visit (i.e., main problem) as well as any additional problems/issues that come up during the visit (i.e., other problem 1, 2, 3, and 4). An opioid-related ICD code as the main problem indicates that the patient was admitted with an opioid-related harm as their primary concern for the hospital visit. An ICD code of interest as other problem 1, 2, 3 or 4 indicates that the patient was admitted to the hospital for some other concern but was found to have an ‘other problem’ related to opioids (specifically, related to opioid poisoning or an OUD).

### 3.1 Overall opioid-related harms

**FIGURE 2:**
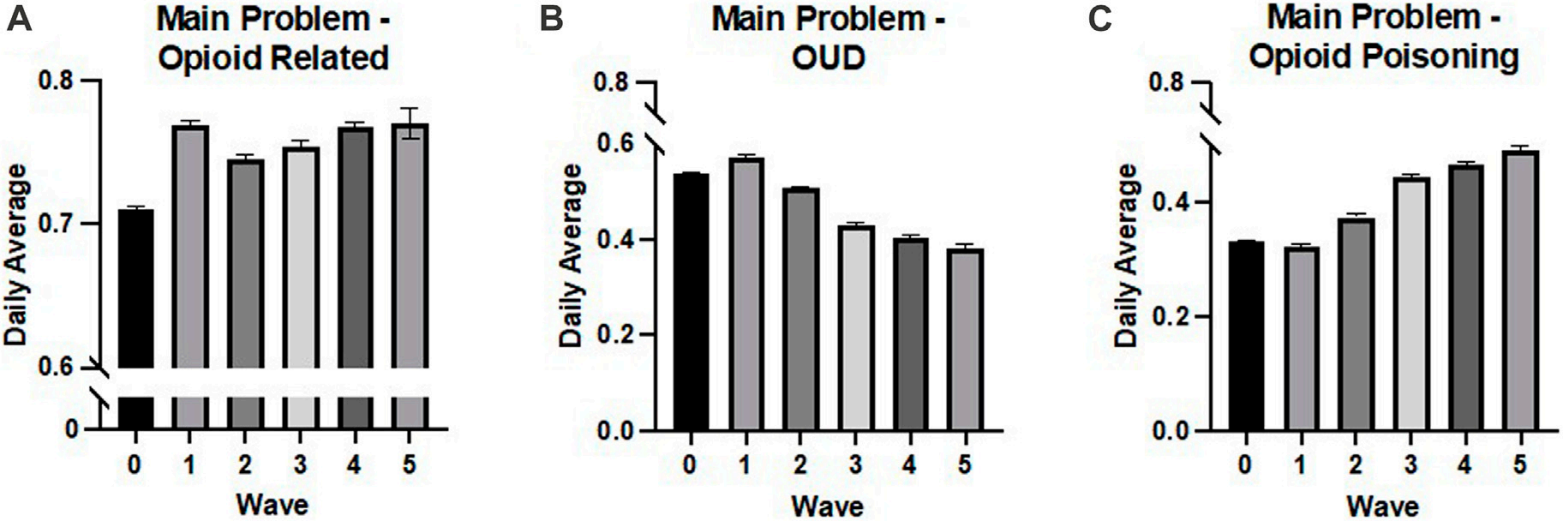
National daily averages from NACRS Data by wave. **(A)** The rate of patients presenting in the ER with an opioid-related main problem significantly increased with the progression of the waves of the pandemic (adj. R^2^ = 0.094, *p* < 0.001, β = 0.306), **(B)** the rate of patients presenting in the ER with OUD as the main problem significantly decreased with the progression of the waves of the pandemic (adj. R^2^= 0.192, *p* < 0.001, β = −0.438). And **(C)** the rate of patients presenting in the rate of patients presenting in the ER significantly increased with the progression of the waves of the pandemic (adj. R^2^ = 0.251, *p* < 0.001, β = 0.501). All values are expressed as means + SEM.

**FIGURE 3:**
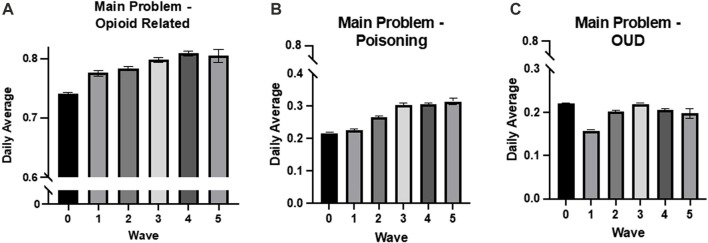
Ontario daily averages from NACRS data by wave. **(A)** The rate of patients presenting in the ER with an opioid-related main problem significantly increased with the progression of the waves of the pandemic (adj R^2^ = 0.121, *p* < 0.001, β = 0.348), **(B)** the rate of patients presenting in the ER with opioid-related poisoning as the main problem significantly increased with the progression of the waves of the pandemic (adj R^2^ = 0.235, *p* < 0.001, β = 0.485), and **(C)** the rate of patients presenting in the ER with OUD as the main problem significantly decreased with the progression of the waves of the pandemic (adj R^2^ = 0.007, *p* < 0.001, β = −0.086). All values are expressed as means +SEM.

Overall, the rate of patients presenting in the ER with any opioid-related main problem significantly increased with the progression of the waves of the pandemic nationally (adj. R^2^ = 0.094, *p* < 0.001, β = 0.306) as well as in Ontario (adj R^2^ = 0.121, *p* < 0.001, β = 0.348; see Figures 2A, 3A). Moreover, the implementation of CERB was associated with a significant increase in patients presenting with an opioid-related main problem nationally (adj. R^2^ = 0.022, *p* < 0.001, β = 0.152) and in Ontario (adj R^2^ = 0.005, *p* = 0.005, β = 0.068). In Ontario, the severity of public health stage was also significantly associated with patients presenting with an opioid-related main problem (adj R^2^ = 0.051, *p* < 0.001, β = 0.228).

The average number of daily visits is also relevant for understanding how opioid-related harms changed in response to the different waves and stages of the pandemic. The rate of patients presenting in the ER for any opioid-related reason was strongly positively associated with the waves of the pandemic in Ontario (adj. R^2^ = 0.730, *p* = 0.019, β = 0.885). The average number of daily visits was not significantly associated with national waves or public health stages in Ontario (*p* = 0.096 and *p* = 0.830, respectively).

### 3.2 Opioid-related poisonings

#### 3.2.1 National (Ontario, Alberta, Nova Scotia, New Brunswick, Yukon, and PEI)

National rates of opioid-related poisonings in the ER significantly increased with the wave of the pandemic (adj. R^2^ = 0.233, *p* < 0.001, β = 0.484). The implementation of CERB, however, was negatively associated with national rates of opioid-related poisonings, indicating a decrease in recorded opioid-related poisonings in the ER nationally during the period when CERB payments were being made (adj. R^2^ = 0.004, *p* = 0.004, β = −0.069).

In addition, the rate of opioid-related poisonings being the main problem for patients presenting to the ER significantly increased with waves of the pandemic (adj. R^2^ = 0.251, *p* < 0.001, β = 0.501; see Figure 2B). The implementation of CERB was negatively associated with the main problem of the patient being an opioid-related poisoning (adj. R^2^ = 0.005, *p* = 0.002, β = −0.073).

#### 3.2.2 Ontario

Rates of opioid-related poisonings in the ER in Ontario increased significantly with the waves of the pandemic (adj R^2^ = 0.131, *p* < 0.001, β = 0.363), severity of public health stage (adj R^2^ = 0.102, *p* < 0.001, β = 0.321), and implementation of CERB (adj R^2^ = 0.038, *p* < 0.001, β = 0.197).

Moreover, the rate of opioid-related poisonings being the main problem for patients in the ER significantly increased with waves of the pandemic (adj R^2^ = 0.235, *p* < 0.001, β = 0.485; see Figure 3B) and public health stages (adj R^2^ = 0.078, *p* < 0.001, β = 0.280). There was not a significant association between CERB and the main problem of the ER visit being an opioid-related poisoning (*p* = 0.396).

### 3.3 Opioid use disorders

#### 3.3.1 National (Ontario, Alberta, Nova Scotia, New Brunswick, Yukon, and PEI)

Nationally, rates of OUDs in the ER significantly decreased with the waves of the pandemic (adj. R^2^ = 0.115, *p* < 0.001, β = −0.339) but significantly increased with the implementation of CERB (adj. R^2^ = 0.005, *p* = 0.003, β = 0.072).

The national rate of OUDs as the main problem in the ER was also negatively associated with the wave of the pandemic (adj. R^2^ = 0.192, *p* < 0.001, β = −0.438; see Figure 2C) and positively associated with CERB (adj. R^2^ = 0.017, *p* < 0.001, β = 0.132).

#### 3.3.2 Ontario

Rates of OUDs in the ER in Ontario significantly decreased with the waves of the pandemic (adj. R^2^ = 0.115, *p* < 0.001, β = −0.339), severity of public health stage (adj. R^2^ = 0.088, *p* < 0.001, β = −0.298), and implementation of CERB (adj. R^2^ = 0.035, *p* < 0.001, β = −0.189).

Rates of OUD as the main problem for ER visits in Ontario also had a significant negative association with the wave of the pandemic (adj R^2^ = 0.007, *p* < 0.001, β = −0.086; see Figure 3C), the severity of public health stages (adj R2 = 0.043, *p* < 0.001, β = −0.208), and CERB (adj R2 = 0.071, *p* < 0.001, β = −0.267).

### 3.4 Opioid-related poisoning and opioid use disorder

#### 3.4.1 National (Ontario, Alberta, Nova Scotia, New Brunswick, Yukon, and PEI)

The national rates of individual patients having *both* an OUD and an opioid-related poisoning concern during the same ER visit had a significantly positive association with the wave of the pandemic (adj. R^2^ = 0.120, *p* < 0.001, β = 0.347) and was not significantly related to the implementation of CERB (*p* = 0.785).

#### 3.4.2 Ontario

In Ontario, rates of individual patients having both an OUD and an opioid-related poisoning concern during the same ER visit significantly increased with the waves of the pandemic (adj R^2^ = 0.049, *p* < 0.001, β = 0.223), severity of public health stage (adj. R^2^ = 0.041, *p* < 0.001, β = 0.205), and implementation of CERB (adj. R^2^ = 0.006, *p* < 0.001, β = 0.080).

### 3.5 Semi-structured interviews

#### 3.5.1 Treatment

##### 3.5.1.1 Demand for services

Many services were strained during the pandemic due to increased demand. One peer support worker described a substantial increase in attendance at meetings for individuals supporting a loved one with an OUD during the COVID-19 pandemic. The peer support program executive director, harm reduction program coordinator, and women’s shelter employee described the increase in demand for mental health and addiction services during the pandemic, including peer support services and harm reduction services. Finally, the women’s shelter employee described a delay in EMS services during the pandemic.

*“My program exploded during COVID, because there was such limited access to […] harm reduction services and […] care and treatment and supplies.”* (harm reduction program coordinator)

*“In 2020-2021, it was a lot. Like 5 to 15 people a [peer support] group. Now, it is much smaller, I am not 100% sure why, but it’s more like, honestly, 2 to 5 […] so during COVID it really increased.”* (peer support program executive director)

*“I know that we were definitely seeing an increase in overdoses at my shelter during COVID […] I know that ambulances would take a long time to get to us for example, but I don’t know if that was because of COVID […] But yeah, I would definitely say that the access was really hard for them to get during that time.”* (women’s shelter employee)

##### 3.5.1.2 Ongoing structural issues

Several service providers discussed components of opioid treatment that were dysfunctional before the onset of the pandemic and were only made worse by upheaval due to the pandemic. For example, the peer support program executive director discussed the lack of coordination between detox centers and residential treatment. The participant explained that patients will be left to live outside of a treatment center for weeks at a time after going through detox, which puts them back into an environment with very little support and a lot of access to substances, without their previous physical tolerance to help prevent fatal overdose. This can result in significant increases in opioid-related poisonings and deaths and has been exacerbated during the COVID-19 pandemic.

##### 3.5.1.3 Service shutdowns and capacity restrictions

Participants also discussed the shutdown of services due to COVID-19, and how this impacted treatment for individuals who use opioids. Participants described a variety of services that shut down for periods of time and/or reduced their capacity significantly.

*“I think for a period of time, when everything was shut down, absolutely, there were more people that had to use on the street, who would normally access the supervised consumption site.”* (peer support program executive director)

*“A whole bunch of […] places, they had to […] change their facilities so they might have had six booths and they went down to two.”* (peer support program executive director)

*“Even private rehab centers like Bellwood had to, any residential treatment place had to put in COVID restrictions.”* (peer support program executive director)

*“One of the things that was really detrimental was the RAAM clinic […] no longer being treated as this urgent service, and no longer was rapid or accessible and still continues, in my opinion, to not be rapid or accessible […] a lot of the in-house services that would have been available […] addiction wise, whether it was counselling or addiction medicine […] pulled a lot of their satellite services out of high needs areas.”* (harm reduction program coordinator)

*“There would be long waitlists, and then by the time you get to the waitlists, it’s you know, with addictions, you’re kind of in a different point at that, especially because addiction is kind of a survival method on the streets, right? So, it’s like, yeah that was definitely a big thing, having those kind of like safe sober beds getting reduced was huge.”* (women’s shelter employee)

The peer support program executive director and women’s shelter employee mentioned that residential treatment centers already had significant waitlists which worsened due to social distancing and isolation measures during the pandemic. Moreover, multiple participants mentioned the impact of restrictions on public indoor spaces on individuals without a home and individuals who use those spaces to socialize and as a safe space to use opioids.

“*It depletes overall health and wellness because now [individuals without a home] are outside 24/7. They can’t even go into a Tim Hortons.”* (harm reduction program coordinator)

*“I think that […] the women just felt more isolated from COVID, because there wasn’t anything open […] like a lot of them would use bathrooms with safe injection sites in it and like needle dispose, but like bathrooms weren’t […] open to the public, so they would be on the streets, so of course unsafe use.”* (women’s shelter employee)

Finally, one participant mentioned that the pandemic impacted access to important harm reduction supplies, such as needles and kits.

*“We were like running low on like supplies for like safe injection sites, like that was a thing that we had trouble getting them, and so like that was not ideal.”* (women’s shelter employee)

##### 3.5.1.4 Isolation

Isolation of individuals who use opioids has also been a major impact of the pandemic according to the service providers who were interviewed. The harm reduction program coordinator talked about the importance of “prosocial contact” and how the lack of prosocial contact due to physical isolation during the pandemic “exacerbated mental health issues” among individuals who use opioids. Moreover, another participant talked about the lack of physical contact and emotional support when meetings moved online, removing an integral part of meetings that support individuals with OUDs.

*“Well, it’s certainly affected meetings from going in-person to going online, that’s been a huge impact, for a lot of clients. It’s caused further isolation; isolation just promotes addiction and mental health problems.”* (peer support worker)

By contrast, the harm reduction program coordinator mentioned that the pandemic decreased isolation for some individuals who were living alone or on the street and had their lives disrupted by fatal overdoses of friends or changes to their living situation, since they moved into “public spaces like shelters and weren’t using alone anymore.”

##### 3.5.1.5 Willingness to access help

When asked about the willingness of individuals to access help during an opioid overdose, half of the participants did not think that this had been significantly impacted by the pandemic (*n* = 2). However, the harm reduction program coordinator discussed the impact of public health messaging around essential services and emergencies on people dealing with an OUD or an opioid-related poisoning. Individuals dealing with opioid-related concerns were not always sure whether to call for help or go to the hospital due to COVID-19 related rules. The participant specifically talked about how messaging around ERs being overwhelmed and existing stigmatization of OUDs impacted the willingness of PWUD to access services. These same uncertainties were described by another participant as resulting in the reuse of drug supplies because individuals using opioids were sometimes unsure whether getting new supplies was an essential service.

*“There was definitely a lot more people reusing supplies, because before they found out about us, they weren’t willing to go downtown because the overwhelming message was don’t leave your home, don’t leave your home, don’t travel unless it's necessary, and that stigma of like what’s necessary […] that message in society is not that this is necessary […] people don’t feel like they’re worthy of that necessary essential service at the best of times, and here we are in a pandemic.”* (harm reduction program coordinator)

*“Somebody said to me, I didn’t even know if I should even call the paramedics because it seems like the healthcare system was […] overwhelmed, and we don’t even know if they would come inside […] the overwhelming message we were hearing was don’t go to the emergency room unless it’s actually urgent, and someone may then second guess whether or not their overdose was urgent.”* (harm reduction program coordinator)

##### 3.5.1.6 Specific wave

When asked about waves of the pandemic, it was difficult for participants to pinpoint specific waves and policies that had the most significant impact on opioid use and opioid-related harms. However, the harm reduction program coordinator said that there were “barricades everywhere to everything” in the first six to 8 months of the pandemic, and that this time had the most significant impact. The same participant talked about the impact that this period had on individuals going through methadone or suboxone treatment, who went from seeing their addiction medicine doctor “weekly, or biweekly, or monthly” to not seeing their doctors for 6 months (or more) and having “their prescription […] rolled over month after month after month”. The participant explained that for individuals who were stable and had been on OAT for a long time, this was sometimes a welcome change, but that for others that were in the middle of reducing or increasing dosages, the lack of contact with their physicians was very difficult. The same participant also talked about the frustration with the removal of methadone restrictions and guidelines during the initial waves and shutdowns of the pandemic, since there were “all these restrictions and guidelines […] which were restrictive for patients” that were suddenly removed. This raised a lot of questions around how regulations for individuals who use opioids are determined and created further frustration that patients were not consistently being consulted on what worked best for them.

##### 3.5.1.7 Adaptation to COVID-19

Although there were many reductions to treatment capacity and availability during the pandemic, participants also discussed services that did not change significantly or that adapted, and may have even improved, during the pandemic. The peer support program executive director discussed Rapid Access Addiction Medicine Clinics (RAAM) and said that “they have continued to expand” and that this is positive, since it is a space where “people can get stigma-free care.” The same participant also mentioned that RAAM clinics “went significantly online.” Other support services also increased their modes of delivery. For example, the peer support program executive director described their organization adding a phone line and increasing the frequency and geographical reach of support groups due to the movement of group meetings online.

*“What did change, was the group support […] when we went into the pandemic, we had like a group meeting […] once or twice a month, and so it was in person. And that we took online, and so now we have four support groups in a month […] online. And it can be anybody across Canada.”* (peer support program executive director)

Finally, the harm reduction program coordinator mentioned that their agency did not shut down, but instead adapted to have services offered outdoors instead of indoors, and that more people discovered and started to access their services in the suburbs, since they were no longer traveling downtown for harm reduction services due to restrictions on movement during the pandemic.

A similar perspective was reiterated more generally from the peer support worker, who described the high degree of adaptation of meetings that went “online quite quickly”. Moreover, the peer support program executive director described the increased flexibility for carries for suboxone and methadone as a “really good thing.” The same participant described the importance of the National Overdose Response Service (NORS) in responding to the pandemic, in addition to virtual support and apps that “should have existed pre COVID” and that were “all really positive and really good.” Finally, the peer support worker mentioned that they had not seen “any issues with the safe injection sites” during the pandemic and that numerous services that were already done over the phone were not impacted.

It was noted by the harm reduction program coordinator, women’s shelter employee, and the peer support program executive director that transitions to virtual resources were generally positive but that the most marginalized individuals struggled to access online and phone services due to a lack of the necessary technology. These changes were particularly impactful to individuals who were accessing low barrier services.

*“I don’t think enough of that was happening to compensate for people that were street homeless and don’t have phones […] which then led to isolation and led to people using more on their own”.* (harm reduction program coordinator)

*“Then the virtual thing was just really, it was a huge barrier for a lot of people just getting services […] the waitlists for in-person appointments that was like six months long because of COVID, and before that we had like a three-week waitlist.”* (women’s shelter employee)

*“We completely shut down in person services, so people that were, it was subsidized so we had a lot of like addictions and stuff like that and accessing it, they couldn’t access it virtually or on the phone because they didn’t have the finances or the education really to be doing that, so I would say when we moved everything virtual that really impacted pretty much everything.”* (women’s shelter employee)

#### 3.5.2 Social supports

##### 3.5.2.1 Financial support

Interviews with service providers revealed that some social services were insufficient before COVID-19, some that were meant to alleviate harms caused harm instead, and others were taken away altogether. In interviews with the harm reduction program coordinator and the peer support program executive director, CERB was identified as a source of harm for some individuals in active addiction. The concern was that CERB was provided in monthly sums that were more likely to be diverted into substances for individuals struggling with an opioid disorder that did not have sufficient supports in place. The peer support program executive director also expressed concern over the fact that CERB was rolled out in such a way that many individuals did not fully realize how the money would later be taxed and that recipients who were not eligible may have to pay it back. This created financial and substance-related issues for some individuals with an OUD.

*“Everyone was getting this CERB money so people had access to all of this disposable income and in the middle of their […] substance use […] that just equates disaster.”* (harm reduction program coordinator)

##### 3.5.2.2 Prisons

Some of the social supports that decreased during COVID-19 were supports for individuals in the carceral system. One participant described some prisons opening their doors and releasing people with “untreated addiction or mental illness” who had nowhere else to go.

*“If they are in prison, they’ve got a roof over their head and they’ve got food. If they’re on the street and it’s COVID, you know, it was like, well the prisons don’t want them, and […] so where are they going to go, the shelters? Well, a lot of them were afraid to go—I mean, shelters aren’t safe places, you know, like, let’s get real, they aren’t. So, they didn’t want to go there, so you know, more homeless, more, all of that.”* (peer support program executive director)

##### 3.5.2.3 Housing and food

The peer support program executive director and peer support worker mentioned the impact of COVID-19 on food banks, saying that “the food bank limited their hours a little bit” during the pandemic and that “a lot of the foodbanks either […] shut down or minimized their access,” demonstrating that access to food may have been impacted by the pandemic. Another participant talked about the impact of the pandemic on fast-food restaurants and how this had a significant impact on individuals facing homelessness and SUDs.

*“Having like the food places shut down for example, or the drive throughs only, like people without cars, so the people with addictions who may not have the financials for the cars, homelessness, can’t just run into Tim’s anymore and buy like the you know kind of affordable bagel, they now are kind of out of luck for a lot of food, right?”* (women’s shelter employee)

Moreover, the peer support program executive director said that housing is essential to addressing the opioid crisis and that the lack of housing was a significant vulnerability to public health changes during the COVID-19 pandemic.

#### 3.5.3 The drug supply

##### 3.5.3.1 Increased toxicity

The toxicity of the drug supply was identified as a significant issue that was worsened by the COVID-19 pandemic. The peer support program executive director and peer support worker discussed the importance of implementing more safe supply programs to deal with the unpredictability and toxicity of the drug supply, especially within the context of the ongoing pandemic where treatment services and harm reduction programs have been adversely impacted. The peer support worker said that “the supply has never been higher risk than it is now.” The same participant discussed the possible links between the changes to supply chains during the pandemic and how this could have resulted in new additives and substances within the illicit supply that were more dangerous, since the “supply got worse and more tainted” during the COVID-19 pandemic. The harm reduction program coordinator reiterated that “the actual supply chain got disrupted” and that the loss of drug testing services and reliable dealers also impacted the safety of illicit opioid use. Another participant talked about some of the experiences of individuals at their shelter with opioids during the pandemic.

*“Sometimes [the individuals at the shelter who overdosed] would be like you know yeah it was fentanyl which kind of made sense, but other times it would be like I got it off this guy, it’s supposed to be clean like this is not as much as they normally are doing, so if they’re doing less than they’re normally doing and they’re overdosing it’s typically a sign that there’s probably something else in it.”* (women’s shelter employee)

One participant described “really bizarre overdoses”, where individuals that were expecting to take fentanyl were exhibiting inconsistent symptoms while experiencing an overdose.

*“People who were drug testing or had reliable sources […] or dealers went out of business […] the actual supply chain got disrupted, so I’d definitely say like really bizarre overdoses, […] what is in the fentanyl that is causing that weird overdose?”* (harm reduction program coordinator)

*“People who said like I had a dealer, I trusted that dealer, you know, they were pretty reliable, the fent was always kind of like this, […] their dealer was like take it easy with this I know it’s really strong or you know this has got some benzo in it be careful, and all of that stuff kind of went away because the supply chain got disrupted.”* (harm reduction program coordinator)

Finally, the same participant mentioned that there was “a lot more coke use” but that this could have been due to more “chaotic” substance use or being “cut off” from fentanyl for periods of time due to supply chain disruptions.

##### 3.5.3.2 Benzodiazepines

Benzodiazepines were brought up as a recent addition to the illicit opioid market that has created massive and scary impacts on individuals that use illicit opioids, such as increased mortality and amnesia. For example, the peer support program executive director described the impacts of benzodiazepines in the opioid supply as creating “full blown amnesia,” an effect that the participant “had never seen […] before.” The same participant described that “people are getting addicted to benzos without even knowing” because they are using illicit opioids. The harm reduction program coordinator and women’s shelter employee commented on increased opioid-related poisonings due to the increased unpredictability and toxicity of the illicit opioid supply during the COVID-19 pandemic.

#### 3.5.4 Opioid-related harms

##### 3.5.4.1 Increased mortality among youth

During interviews with service providers, young people were described as suffering disproportionately from opioid-related harms during the pandemic. The peer support program executive director talked about how the parents that they support who are caring for someone with a substance use disorder “lost minor children” while waiting for services. The same service provider mentioned that “up to a third of those people haven’t been diagnosed with an opioid use disorder,” referring to people that are fatally overdosing. The participant suggested that this could mean that the rise in opioid-related overdoses in young people is not necessarily because of increases in OUDs, but due to overdoses in young people that are using opioids recreationally. The peer support worker also mentioned that laws that protect privacy often prevent parents from intervening in their children’s opioid use treatment, and sometimes even result in the parents not knowing that their child is using opioids or has overdosed in the past. Finally, one participant argued for the importance of regulating the drug supply to protect children who may be gaining access to opioids.

*“I think the best way to protect [kids] is, again, to regulate it for adults […] knowing that then the kids, hopefully, who decide to use will get regulated substance, because if they’re opioid naive and it’s got fentanyl, not, you know, heroin, and it’s not regulated, that I think is the reason for that exponential growth, and making it the number one cause of death.”* (peer support program executive director)

##### 3.5.4.2 Opioid use disorders

In addition to the increase in opioid-related deaths among young people, the peer support program executive director, peer support worker, and women’s shelter employee described the prevalence and severity of OUDs as having increased significantly due to COVID-19, exacerbated by isolation and a lack of services. The harm reduction program coordinator talked about a “spike early on” in OUDs, and that generally they would have thought that OUDs would have increased during the pandemic, but that there were some instances in which they had clients’ substance use decrease due to the pandemic.

*“I think of a few of my clients, who, because of COVID and maybe some fatal overdoses that have happened around them, […] they’ve had to move, you know like they went from having a semi-stable place to live to being in shelter, to being here, to being there, and in some cases that chaos has actually helped to stabilize them, because all of the sudden using became a bigger problem, so survival wise, having a roof over their head and managing that day-to-day stuff became more important than their substance use so they’re still using, but they’re using less.”* (harm reduction program coordinator)

*“It [opioid use disorders] got a lot worse […] our numbers actually were spiking because of the pandemic, of people accessing our shelter, like we were doubling, and […] the government actually gave us funding to be open during the summer as well, because they were seeing the need for these women to have the safe beds, and then from the transition from winter to summer like our numbers stayed the exact same which was kind of unheard of from before.”* (women’s shelter employee)

*“I guess they were saying at this [safe injection site] they were seeing women like inject it in their neck, their jugular, just injecting it in really weird places that weren’t as common and from my understanding, like you know, it’s because, it just gives you more of an intense high, and so I guess the other places were being overused […]so I guess maybe the frequency of use maybe is going up, because people are getting a little bit more creative with where they’re injecting it.”* (women’s shelter employee)

##### 3.5.4.3 Women

The women’s shelter employee was able to shed light on some of the gendered impacts of the COVID-19 pandemic on the opioid epidemic, and how vulnerabilities significantly increased among women for reasons related to the pandemic and OUDs.

*“I think one thing that I had a really big problem with, was when people, well you remember at the beginning of COVID when it was really like one person should be going out, no one should be going in stores, I just feel like from like a safety perspective that was very odd to me, because especially people facing homelessness and you know homelessness and addiction a lot of time is hand in hand, they, especially women would be in pairs and be, would be going in pairs for safety reasons right because there’s the risk of human trafficking […] on the streets, the men know who the vulnerable women are and all that kind of stuff, so when women were going in pairs everywhere and maybe going to stores in pairs and all that, like they were getting a lot of shame for that.”* (women’s shelter employee)

*“We noticed that there was a lot of men driving around and would get these women to come into their vehicles, get them high, and then would try to do these things or try to literally kidnap them essentially for human trafficking, and that kind of happened near the end, and I’m just kind of adding things up in my head, and these women are, they kind of became more vulnerable during the pandemic, and during COVID and through their addictions, and these men were coming with money […] and were exploiting these women now.”* (women’s shelter employee)

#### 3.5.5 Stigma

Stigma is a complex social phenomenon that presents real barriers to treatment and services for individuals with a SUD. The peer support program executive director said that due to the increase in opioid-related harms during the pandemic, it is possible that stigma has gotten better because “the numbers are getting so bad and it’s effecting so many families, including families with young children.” By contrast, the same participant pointed out the “hypocrisy of society” with regards to the rapid and forceful response to COVID-19 compared to inadequate interventions into the opioid crisis, and that this is evidence of the stigma that continues to harm individuals that use opioids.

*“The massive stigma […] prevents a proper […] response to the opioid crisis, a lack of resources, a lack of evidence-based treatment, a lack of everything.”* (peer support program executive director)

Another participant talked about how the increased visibility of homelessness and addiction during the pandemic, due to worsening SUDs and fewer services, contributed to increased stigma among the general public.

*“I would maybe think [stigma] got worse just because the rates of overdose was going up and then like people that use addictions were also facing homelessness, had less places to go, so they were, they were outside more, right, and I think that definitely people were not very kind to that, kind of seeing them more, a little bit more prevalent I guess for a little bit […] that it might have added to the stigma in a negative way.”* (women’s shelter employee)

Finally, the peer support worker and harm reduction program coordinator did not think that the pandemic has a significant impact on stigma, saying that it has not helped, but has not made it worse.

#### 3.5.6 Recommendations

##### 3.5.6.1 Treatment

The peer support program executive director made several suggestions for how treatment services could have been improved during the pandemic to alleviate opioid-related harms. For example, they mentioned the importance of having “even more RAAM clinics” and suggested that they “expand their hours.” Moreover, the same participant mentioned that individuals showing up in the ER with an opioid-related issue need to be screened to see if they are “a recreational user who overdosed” or if they are in the ER because “they can’t stop using.” Service providers received an impression from their clients of a lack of resources and time for treating non-emergent opioid-related issues in the ER during the pandemic, such as OUDs, and expressed a need for more robust mechanisms of referral to addiction services and medication treatments for individuals with an OUD who end up in the ER. In addition to screening individuals in the ER for an OUD, the participant talked about the importance of educating “the medical community about what addiction is;” specifically, that it is a disease and not a moral failing. The participant also suggested that addiction medicine should be more integrated into primary care. Finally, the participant talked about how the transition between detox and residential treatment needs to be “seamless,” and that this is crucial for protecting individuals who use opioids in the future.

*“The biggest thing we could do is fix the system as soon as possible, so when we’re going through any future wave, people have the support that they need to be well and stay alive.”* (peer support program executive director)

The harm reduction program coordinator talked about the importance of treating addiction medicine and intersecting social services as essential, including food services, drop-in access, and residential treatment centers. Moreover, the participant described how COVID-19 “panic overrode measuring risk,” resulting in a massive loss of treatment services for individuals who suffered without them. The same participant urged the implementation of “more community-based services” and the reimplementation of services to pre-COVID-19 standards, including in-person drop-in spaces and normal hours of operation. Finally, another participant emphasized the importance of housing, in conjunction with other treatment programs, to allow individuals to address their OUD.

*“A lot of people who are experiencing homelessness are also experiencing addiction […] once people have housing, they can start getting clean, they can start focusing on other kind of stuff, because housing is an essential, right? And without that that’s how you start falling into things like addictions. I think we need to start targeting the reasons why people are using addictions […] at a systematic level, start you know addressing the poverty.”* (women’s shelter employee)

##### 3.5.6.2 Safe supply

Safe supply was discussed extensively by the peer support program executive director and peer support worker as an important and effective method for decreasing opioid-related harms. The peer support program executive director specifically advocated for drugs to be regulated “in accordance with their harm.” The same participant described the fact that “it is not possible to exercise agency with respect to how much you’re getting” when using an illegal substance. The peer support worker talked about how their organization was working towards developing an approach for safe supply to be presented to policymakers.

*“The bottom line is we need to make sure that everybody who uses illegal substances has access to a regulated supply of that substance, whether they have recreational use or they have problematic use.”* (peer support program executive director)

## 4 Discussion

### 4.1 Factors related to increased recordings of opioid-related poisonings

The significant association between opioid-related poisonings and the waves of the pandemic, both nationally and in Ontario, indicates that the impact of the COVID-19 pandemic on the opioid epidemic increased over time. Moreover, the stronger association between opioid-related poisonings being the main problem for ER visits and the waves of the pandemic, both nationally and in Ontario, further suggests that opioid-related poisonings were worsening throughout the waves of the pandemic. Together, this points to a cumulative effect of the pandemic’s restrictions on individuals who use opioids, particularly given the fact that opioid-related mortalities also increased over the same time period (Public Health Agency of Canada, 2022).

The positive associated between patients presenting with both an opioid-related poisoning and an OUD is likely driven by the increase in opioid-related poisonings, since OUDs were negatively associated with all predictors; however, the increasing overlap in opioid-related poisonings and OUDs nationally by wave and in Ontario by wave, stage, and CERB still demonstrates that many individuals were experiencing increased opioid-related harms and that more patients with an OUD were experiencing poisonings. This points to an increased severity of OUDs as the waves progressed nationally, and as public health measures became stricter and CERB was implemented in Ontario.

**FIGURE 4:**
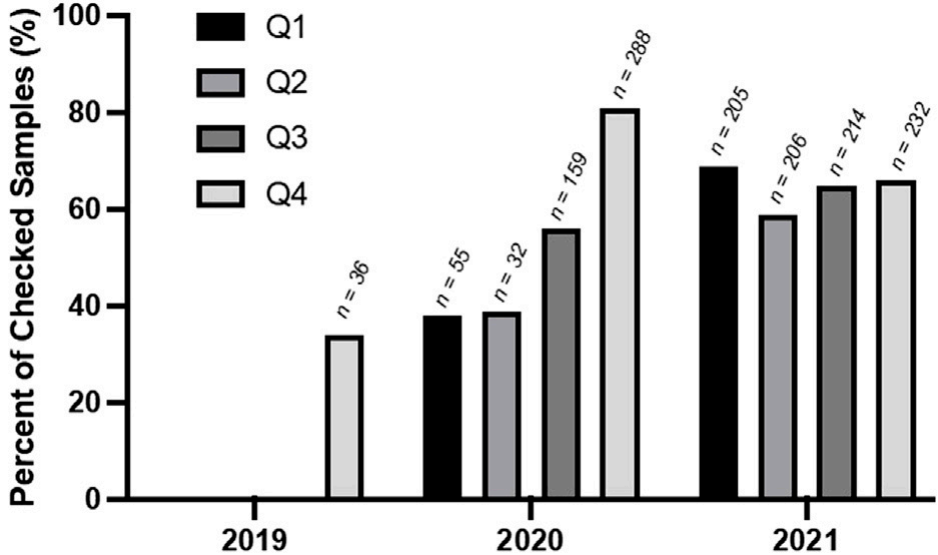
Number of benzodiazepeine-related drug samples expected to be fentanyl between Q4 of 2019 and Q4 of 2021 (adapted from the centre on drug policy Evaluation, 2022). The number of samples checked in each quarter (n) is indicated above each bar.

Based on interviews with service providers, the inaccessibility of treatment services, prolonged loss of important social contact, and increased stigmatization of individuals who use opioids inside and outside the ER could all have had a cumulative impact on individuals dealing with an OUD and led to increased and riskier use. Moreover, for individuals with an OUD or using opioids recreationally, the increased toxicity of the opioid supply could have significantly impacted rates of opioid-related poisonings and opioid-related mortalities. Disruptions in supply chains and shifts in distributors due to border closures and border restrictions resulted in less predictability and new substances in the supply, particularly benzodiazepines (Figure 4). Increased drug toxicity caused by the pandemic was cited as a factor contributing to opioid-related harms by interview participants and is also cited in the literature as a major impact on PWUD (Ali et al., 2021; Centre on Drug Policy Evaluation, 2022; McAdam et al., 2022).

In addition, the significant positive association between opioid-related poisonings in Ontario and the strictness of public health measures is likely connected to the fact that these measures brought about significant changes to capacity restrictions, availability of services, and disruptions to supply chains, all of which were discussed by service providers as sources of opioid-related harms. Given the various intersecting factors that could have contributed to changes to opioid-related harms, the magnitude of the R^2^ value for this relationship indicates that the COVID-19 policy decisions that were instated to combat the spread of COVID-19 had severe impacts on individuals who use opioids. This finding suggests that future public health measures must be further considered for their ability to balance the needs of diverse populations.

### 4.2 Factors related to decreased recordings of opioid use disorders

The negative association between OUDs and the waves of the pandemic nationally and in Ontario contradicts the perspectives of service providers on the rate of OUDs during the pandemic. The negative association between waves and the rate of OUDs as the main problem in ER visits was also stronger nationally than in Ontario. Moreover, the negative association between OUDs and the severity of public health measures demonstrates a decrease in OUD recordings as the severity of the pandemic increased.

Service providers observed indications that OUDs worsened during the pandemic. For example, service providers observed the use of unconventional injection sites due to overuse of other injection sites. Moreover, isolation and restrictions on public spaces deprived individuals looking for support for opioid use or just trying to maintain social networks from crucial prosocial connection, emotional support, and physical contact. Additionally, service providers observed a lack of effective treatment and support resources, especially for the most marginalized who struggled to access virtual services.

Crucially, service providers indicated that their clients were inconsistently offered addiction treatment in the ER. Service providers suggested that their clients often did not receive OUD diagnoses and follow-through on addiction treatment, services, and medication. This suggests that even when individuals with an OUD made it to the ER, they may not have been recorded as having been treated for their OUD.

Finally, participants explained that the strong messaging from public health around the importance of staying at home and only leaving for essential reasons, particularly at the height of public health restrictions, created hesitancy from individuals dealing with opioid use or an OUD to get help. Social perceptions that substance use is a choice and that treatment for substance use is not essential was cited as decreasing the willingness of individuals with an OUD to access harm reduction services and OUD treatment during stay-at-home orders and lockdowns in particular.

All of these factors could help explain the discrepancy between the observations of service providers of worsening OUDs, and the decreased recordings of OUDs in the ER. Crucially, these explanatory factors point to an increase in opioid-related harms, and a decrease in individuals who use opioids accessing the services that they needed and/or being properly treated for their OUD. Not only did ER visits for OUDs decrease, but several studies and service providers found a decrease or insignificant effect of the COVID-19 pandemic on the provision of OAT and social supports (Garg et al., 2022; Kitchen et al., 2022). This overall lack of treatment and support, particularly considering the increasing rate of poisonings, could have contributed to the increase in opioid-related mortalities observed across the country, while the ER was not capturing increased OUDs.

### 4.3 CERB

CERB was brought up by service providers as a source of harm for individuals with an OUD, despite not being specifically brought up by the reviewer.

The negative association between CERB and OUDs and the positive association between CERB and opioid-related poisonings in Ontario suggests that the quantitative data may be reflecting what the service providers talked about. For example, service providers suggested that the disposable income provide by CERB increased the accessibility of drugs and may have caused spikes in opioid-related poisonings, especially when paired with the toxicity of the drug supply. However, the opposite trends were seen nationally, with CERB being positively associated with OUDs and negatively associated with opioid-related poisonings. The magnitude of R^2^ is small for the national data, suggesting that the impact of CERB was minimal. While the magnitude of R^2^ in for Ontario was also small, it still shows that CERB accounted for approximately four percent of the decrease in OUDs and four percent of the increase in opioid-related poisonings, which could have been due to increased access to substances; however, further research is needed on the impact of CERB on PWUD.

### 4.4 Limitations and future research

One of the limitations of this study was the limited sample size of service providers that participated in interviews. Since only four participants were interviewed, the breadth and diversity of experiences and perspectives on opioid use during the pandemic was limited; however, participants offered perspectives from a variety of organizations and various capacities within organizations that offer opioid-related services, and drew on lived experience, observations, and research to answer questions.

In addition, the use of NACRS data limited the national data to the six participating provinces and territories. Within these provinces and territories, only participating hospitals were included in the NACRS data. The results showed a stronger relationship between the wave of the pandemic and opioid-related poisonings nationally compared to in Ontario. This could be due to the presence of other provinces that have been hard-hit by the opioid epidemic in the national dataset, particularly Alberta and other areas in Western Canada; however, British Columbia is a notable missing province in the dataset, since BC is considered the epicenter of the opioid epidemic in Canada (Public Health Agency of Canada, 2022). Although the exclusion of BC means that the national dataset is missing a key province in the story of the opioid epidemic in Canada, the absence of BC further emphasizes the impact of the opioid-related harms on other regions in Canada that are often not the center of conversations on the opioid epidemic in Canada.

Furthermore, ICD codes are being used as a proxy to measure of opioid-related harms. For example, ICD codes are used for billing, which differs between provinces, creating possible uneven biases when it comes to the inclusion of ICD codes and national and international inconsistencies in the use of ICD codes have been identified (*ICD—ICD-10-CM—International Classification of Diseases, ICD-10-CM/PCS Transition)*, 2019; Otero Varela et al., 2021). For the purposes of this study, we were looking at the relative changes in opioid-related harms rather than absolute values, thereby decreasing the possible impact of this bias, but not eliminating it completely.

### 4.5 Recommendations

The COVID-19 pandemic undoubtedly increased opioid-related harms. Opioid-related mortalities skyrocketed, and opioid-related poisonings increased as the waves of the pandemic progressed and as the public health measures in Ontario increased in severity. However, the disparities between service provider accounts of the impact of the pandemic on OUDs and ER records and the overall lack of access to alternative treatment options leads us to a concerning conclusion: individuals living with an OUD experienced more barriers to treatment, greater risk factors for use, and increased danger with use, while accessing treatment and support services less. These findings strongly support the need for improved treatment of long-term substance use concerns in the ER, the treatment of addiction support services as essential services during states of emergency, and the need for action on the toxic and unpredictable drug supply. Service providers have, and continue to, call for safer supply, the prioritization of addiction services as essential, and improved referrals for opioid use treatment in the ER to better support individuals who use opioids and combat the opioid epidemic.

## Data Availability

The data analyzed in this study is subject to the following licenses/restrictions: Data is available upon request. Requests to access these datasets should be directed to mollyhutchinson@cmail.carleton.ca.
